# Bridging Carotenoid-to-Bacteriochlorophyll Energy Transfer of Purple Bacteria LH2 With Temperature Variations: Insights From Conformational Changes

**DOI:** 10.3389/fchem.2021.764107

**Published:** 2021-10-04

**Authors:** Ruichao Mao, Xiaocong Wang, Jun Gao

**Affiliations:** Hubei Key Laboratory of Agricultural Bioinformatics, College of Informatics, Huazhong Agricultural University, Wuhan, China

**Keywords:** photosynthesis, light-harvesting complex 2, molecular dynamics simulation, energy transfer, temperature effect

## Abstract

Photosynthesis is a key process for converting light energy into chemical energy and providing food for lives on Earth. Understanding the mechanism for the energy transfers could provide insights into regulating energy transfers in photosynthesis and designing artificial photosynthesis systems. Many efforts have been devoted to exploring the mechanism of temperature variations affecting the excitonic properties of LH2. In this study, we performed all-atom molecular dynamics (MD) simulations and quantum mechanics calculations for LH2 complex from purple bacteria along with its membrane environment under three typical temperatures: 270, 300, and 330 K. The structural analysis from validated MD simulations showed that the higher temperature impaired interactions at N-terminus of both α and β polypeptide helices and led to the dissociation of this hetero polypeptide dimer. Rhodopin-β-D-glucosides (RG1) moved centripetally with α polypeptide helices when temperature increased and enlarged their distances with bacteriochlorophylls molecules that have the absorption peak at 850 nm (B850), which resulted in reducing the coupling strengths between RG1 and B850 molecules. The present study reported a cascading mechanism for temperature regulating the energy transfers in LH2 of purple bacteria.

## Introduction

Photosynthesis is one of the most important bio-activities on the planet that converts light energy into chemical energy and stores in carbohydrate molecules by plants, algae, and photosynthetic bacteria. Understanding the mechanism of photosynthesis, especially the energy transfers, has drawn great interests in the scientific research community (Sundstrom et al., 1999; Renger and Muh, 2013; Komatsu et al., 2015). Many studies employed photosynthetic bacteria, such as purple bacteria, as the model for energy transfer apparatus in photosynthesis due to the simplicity and symmetry of their light harvesting systems as well as the similarity of their energy transfers to those in plants and algae (Cogdell et al., 2006; Shrestha and Jakubikova, 2015; Sisto et al., 2017; Cupellini et al., 2018).

**FIGURE 1:**
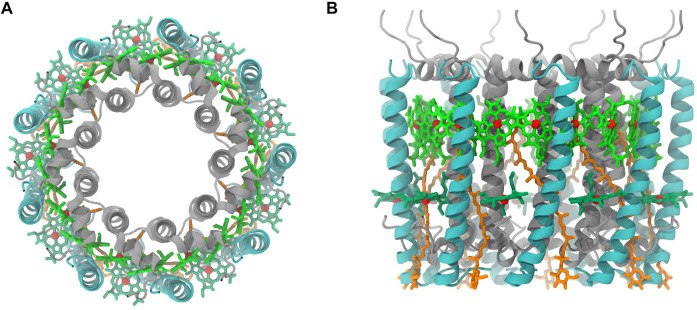
Purple bacteria LH2 complex (**(A)**, top view **(B)**, side view). *α* and β polypeptide helices are color in silver and cyan, respectively. Pigment molecules are colored in dark green (B800), bright green (B850), and orange (RG1). The phytol portion in BChl was not displayed.

In the initial step of energy transfer for photosynthesis process in purple bacteria, light-harvesting complex 2 (LH2) captured energy from light and transferred to light-harvesting complex 1 (LH1), which later passes the harvested energy to reaction center (RC) for photoelectric conversion (Curutchet and Mennucci, 2017; Mirkovic et al., 2017). Thus, LH2 has attracted many attentions in the photosynthesis studies. Most of LH2 complex in purple bacteria has a C_9_-symmetrical ring that is composed of 9 pigments-protein subunits (Figure 1). Each subunit contains a hetero dimeric helical transmembrane polypeptide, in which α and β polypeptide helices locate on the inner and outer side of the ring. Among each dimer of these helices, there are three bacteriochlorophylls (BChl) and a carotenoid molecule, showing in circular arrangements together with the helical dimers. One of the BChl molecules is close to the cytoplasmic side, named B800, that is responsible for the absorption peak at 800 nm, and two other BChl molecules at the periplasmic side, named B850, whose absorption peak is near 850 nm. The carotenoid molecule has an absorption peak at 400–550 nm and a long alkene chain that lies across the trans-membrane helices and interacts with both B800 and B850 molecules (Frank and Cogdell, 1996). After pigment molecules absorbing light energies, RG1 transfers its energies to B800 and B850 molecules, and those energies obtained by B800 molecules will be eventually passed on to B850 molecules to complete the energy transfers within the LH2 system (Mirkovic et al., 2017). On the basis of the established energy transfer pathway, many efforts have been devoted to understand the mechanisms for regulating the energy transfers in LH2, which could provide vital information for designing artificial photosynthesis systems (Mirkovic et al., 2017).

The light-harvesting apparatus in photosynthetic organisms are diverse in order to adapt to different living environments, including temperature variations (Cogdell et al., 2006; Solov’ev and Erokhin, 2008; Ivanov et al., 2017; Madireddi et al., 2019; Croce, 2020). Exploring the mechanisms of temperature adaptation has a profound impact on understanding the energy transfer process of photosynthesis. Studies of higher plants showed that the temperature was one of the regulatory switches for light-harvesting complex of photosystem II (LHCII), which plays an essential role in their light energy harvesting process (Li et al., 2020). Yet, such regulatory mechanisms, if existed, have not been thoroughly studied for LH2 in purple bacteria. Given the generality and exemplariness of LH2 in photosynthesis study, exploring the regulatory mechanisms for energy transfers in LH2 complex has been of great interests. Previous experimental studies showed that LH2 in different species responded to temperature variations differently (Zerlauskiene et al., 2008; Pajusalu et al., 2011; Trinkunas et al., 2012; Shi et al., 2015; Ratsep et al., 2018). Shi et al. showed that in Thermochromatium tepidum, the amplitudes of the B850 absorption band of the LH2 complex displayed a significant reduction as the temperature increased, while the B800 absorption band was nearly unaffected. Yet, in the LH2 complex from Rhodobacter sphaeroides, the amplitudes of both B800 and B850 absorption bands decreased significantly as the temperature increased (Shi et al., 2015). Ratsep et al. (2018) also showed that in the LH2 complex from Rhodoblastus acidophilus, the amplitudes of the B800 and B850 absorption bands also had a significant decrease when temperature increased, suggesting the weakening of the absorbance of the system. Furthermore, the increase of the temperature to 70°C led to the disappearances for the absorption peaks at both 800 and 850 nm and the appearance for a new and dominating absorption peak at 775 nm, which were caused by large-scale deformation of the protein indicated by the Circular dichroism (CD) spectroscopy measurements (Ratsep et al., 2018). Many theoretical studies also showed that temperature could alter the conformations for proteins and surround pigment molecules in LH2, and, in turn, mediate the site energies and electronic couplings between pigment molecules, and eventually affect the light capture capability and energy transfer efficiencies of pigment molecules (Cupellini et al., 2016; Li et al., 2020). Yet, the underlying mechanism of these regulations at the atomic level requires further elucidation. Given the dynamic nature of LH2 complex, it is critical to understand the properties beyond the measurements from static structures. Molecular dynamics (MD) simulations with proper force fields have been widely employed to study dynamic characters of biological molecules in physiological environments. In addition, combining with quantum mechanics (QM) calculations, the electronic nature in the dynamic process of pigment molecules can be explored.

In the present study, the molecular model for purple bacteria LH2 in micro-second MD simulations reproduced structural stability, the experimentally observed essential intermolecular hydrogen bond interactions and absorption spectrum changes under different temperatures. In elucidating the mechanism for temperature-regulated RG1-B850 energy transfer with the molecular model of LH2, the interactions at the N-terminus of *α* and *β* polypeptide helices reduced as the temperature increased, which led to the dissociation of *α* and *β* polypeptide helices. RG1 molecules moved centripetally with *α* polypeptide helices due to their strong interactions in the tail segment of RG1 molecules; on the other hand, B850 molecules that couple and receive energies from RG1 molecules remained steady when temperature increased, and caused larger distances between RG1 and B850 molecules. The electronic energy transfer (EET) analyses in QM calculations demonstrated that this increased distances due to the increased temperature diminished the coupling strength between RG1 and B850 molecules and decreased the energy transfer capacity in purple bacteria LH2 system.

## Computational Methods

### Model Generation and MD Simulation Setup

The initial coordinates of the LH2 complex were obtained from the crystal structure of Rhodoblastus acidophilus (PDB entry code: 1NKZ) (Papiz et al., 2003). Molecules that are not protein or pigment molecules were removed before molecular models were built. In addition, a second set of RG1 molecules was also removed as there are still debates regarding their existence (Gall et al., 2006). This crystal structure only contains one third of a ring. The complete ring of LH2 system were reconstructed according to crystal cell information. The valence parameters for protein residues including carboxyl-α-Met1 (CXM) were from ff14SB force field parameters (Maier et al., 2015), those for B800 and B850 molecules as well as their atomic partial charges were derived by Ceccarelli et al. (2003), and those for RG1 molecules was taken from generalized AMBER force field (GAFF) (Wang et al., 2004). The AM1-BCC atomic charges (Jakalian et al., 2002) for CXM and RG1 molecules were derived with Antechamber module in AmberTools14 (Case et al., 2005). The molecular model for LH2 was generated by tLEaP module in AMBERTools14 with all histidine residues kept in ε configuration except those axially coordinated with the B850. Then, the LH2 system was embedded into a lipid bilayer composed by 2-oleoyl-1-palmitoyl-sn-glycero-3-phosphocholine (POPC) built with Membrane Plugin in VMD (Humphrey et al., 1996), in which the force field parameters for POPC were derived by Kasson et al. (2010).

Two layers of TIP3P water molecules were added to each side of the hydrophilic parts of POPC with a thickness of 25 Å. The LH2 complex was added to the center of the solvated bilayer with the axes of helices in α and β chains parallel to the normal vector of membrane. All water and POPC molecules within 0.8 Å of any atom of LH2 complex were removed. Lastly, the counter ions were added to achieve neutralization by using the tLEaP module. The solvated complex with lipid bilayer was shown in Supplementary Figure S1.

The energy minimization of the solvated complexes was performed for 7,000 steps with conjugate-gradient, in which restraints (10 kcal/mol∙A^2^) to all non-hydrogen atoms in proteins and pigment molecules were only applied in the first 5,000 steps. Then, the entire system was heated to 270, 300, 330 K individually at a unifying speed of 400 fs/K with a 2 fs time step under nVT condition and the same restraints in the previous step. After heating, each system was equilibrated a smaller restraint (2 kcal/mol∙A^2^) under nVT and nPT conditions, consecutively, and each for 2 ns with a time step of 2 fs. Prior to data collection, each system was equilibrated for 5 ns with all restraints removed and a time step of 1 fs under nPT condition. Data collections were carried out with a time step of 2 fs for a total of 1 μs under each temperature.

Covalent bonds involving hydrogen atoms were constrained using SHAKE algorithm (Miyamoto and Kollman, 1992). A nonbond cutoff of 10 Å was applied to van der Waals interactions and long-range electrostatics were treated with the particle mesh Ewald approximation with a grid spacing of 1 Å (Essmann et al., 1995). The temperature was maintained by employing a Langevin thermostat with a damping coefficient of 5 ps^−1^. All MD simulations were carried out at constant pressure of 1 atm by using the Nosé-Hoover Langevin piston method with a decay period of 100 fs and a damping timescale of 100 fs.

### Ring Fitting

A ring was fitted to the circle formed by pigment molecules and proteins by selecting a periodic point per pigments-protein subunit. The center of the fitted ring was first randomly chosen to calculate $\Delta R^{2}$ :

$$\begin{array}{c} \Delta R^{2}=\overset{9}{\underset{i=1}{\sum }}{\left(r_{i}-\bar{r}\right)}^{2} \end{array}$$ (1)

where $\bar{r}$ is the average value of 9 $r_{i}$ in a single structure of an MD simulation trajectory. Then, the center of the fitted ring was determined by minimizing $\Delta R^{2}$ with the least-squares minimization method. The $\bar{r}$ for the determined center was employed as the radius of the fitted ring.

### Absorption Spectrum Calculations

The excitonic states of the complete system can be determined from the site energies and the electronic couplings gained from the QM calculations, for which an excitonic Hamiltonian that includes the site energies (ϵ) for individual BChls and their electronic couplings (V) for the two BChl rings in LH2 is constructed:

$$\begin{array}{c} \hat{H}\;=\underset{n}{\sum }\varepsilon_{n}|n\rangle \langle n|+\;\underset{\mathrm{mn}}{\sum }V_{\mathrm{mn}}|n\rangle \langle m| \end{array}$$ (2)

in which, |n〉 represents the excited state of a single BChl, and the energy $\varepsilon_{n}$ corresponds to the energy difference between its ground state and the excited state; *m* and *n* represents any two different pigment molecules, and $V_{mn}$ is the electronic coupling between *m* and *n*.

The excited state for each BChl molecule was computed with TD-DFT calculations at the CAM-B3LYP/6-31G(d) level of theories in Gaussian16 (Frisch et al., 2016) and it should be noted that the site energies of 9 BChls in the B800 ring was shifted by −0.07 eV to match the experimental peak at 800 nm. The coupling strengths for each pair of molecules were obtained from the point dipole approximation (PDA):

$$\begin{array}{c} V_{\mathrm{ij}}^{\mathrm{PDA}}=\left|\mu_{i}\right|\left|\mu_{j}\right|\frac{\kappa }{R_{\mathrm{ij}}^{3}} \end{array}$$ (3)

where $V_{ij}^{PDA}$ is the coupling strength between any two coupled molecules i and j , whose transition dipole moments were noted as $\left|\mu_{i}\right|$ and $\left|\mu_{j}\right|$ ; $R_{ij}$ is the distance between the two dipoles; κ accounts for the relative orientation between two dipoles:

$$\begin{array}{c} \kappa ={\hat{\mu }}_{i}\cdot {\hat{\mu }}_{j}-3\left({\hat{\mu }}_{i}\cdot {\hat{R}}_{\mathrm{ij}}\right)\left({\hat{\mu }}_{j}\cdot {\hat{R}}_{\mathrm{ij}}\right) \end{array}$$ (4)

where the hat notation indicates the unit vector.

100 snapshots were evenly extracted along the last 100 ns MD simulation trajectories under different temperatures and used to compute absorption spectra. For each LH2 structure containing 27 pigment molecules, the calculated spectra were broadened using a Gaussian window to model inhomogeneous broadening effects (Yin et al., 2007)

$$\begin{array}{c} A\left(v\right)=\frac{v}{2\sqrt{\pi }\sigma }\overset{27}{\underset{i=1}{\sum }}M_{i}^{2}\exp\left[-\frac{{\left(E_{i}-\mathrm{hv}\right)}^{2}}{2\sigma^{2}}\right] \end{array}$$ (5)

in which, *A* is the absorption strength at transition frequency v ; *σ* is the standard deviation of the Gaussian, with a value of 0.022 eV; $E_{i}$ and $M_{i}$ are the energy and transition dipole moment of the *i* ^th^ eigenvector of the previously computed exciton coupling Hamiltonian $\hat{H}$ , respectively.

The final spectrum under each temperature was obtained by bimodal fitting of 100 individual spectra.

### Electronic Coupling Calculations

Electronic couplings were computed with three methods with different level of accuracies, among which the transition densities from the ground to the excited state were treated differently. Firstly, the previously described PDA was computed from the transition dipole moments obtained with TD-DFT methodology at the CAM-B3LYP/6-31G(d) level of theories in Gaussian16. Secondly, a new calculation scheme with numerical integration of the transition density on the spatial coordinates conducted by the BDF software package (Zhang et al., 2020) was utilized to compute the electronic couplings again, in which the excited state was computed with TD-DFT calculations at the CAM-B3LYP/def2-sv(p) level of theories. Lastly, the EET analysis (Iozzi et al., 2004) in Gaussian16 was employed for calculating electronic couplings with the considerations of the solvent effects, which are estimated by using Polarizable continuum model (PCM) (single cavity). The excited states in this scheme were also computed with TD-DFT methodology at the CAM-B3LYP/6-31G(d) level of theories in Gaussian16.

## Results and Discussions

### Structural Variations in MD Simulations Under Different Temperatures

The molecular model of LH2 complex with solvated lipid bilayer in MD simulation was first validated by reproducing its structural properties and interactions. The RMSD values from MD simulations under 270, 300, and 330 K from non-hydrogen atoms in protein backbone, RG1 molecules, and porphyrin ring in B800 and B850 molecules were all under 4 Å (Supplementary Figure S2). Furthermore, both polypeptide helices and pigment molecules in the solvated complex reached and maintained structural stability after approximately 200 ns of MD simulations as seen from their RMSD values. Key hydrogen bonds linking both *α* and *β* polypeptide helices observed in the crystallographic data were reproduced in the MD simulations (Table 1), although those regarding Nε atom in the side chain and N atom in the backbone showed smaller occupancies than other ones. These hydrogen bond interactions were critical to the stability of the complex dimers formed by *α* and *β* polypeptide helices (Arluison et al., 2004; Parkes-Loach et al., 2004; Wang et al., 2005). The representative snapshot, the frame in the simulation trajectory that has the lowest RMSD value referencing to the average geometry from the MD simulation, under 300 K was extracted to compare to the crystal structure. The comparison of these two structures displayed significant similarities in conformations of protein backbone and positions, as well as orientations, of RG1 molecules, or porphyrin ring during the MD simulations (Supplementary Figure S3).

**TABLE 1:** Essential intermolecular hydrogen bond occupancies between residues in α and β polypeptide helices.

α-helices		β helices		Occupancy^a^		
				270K	300K	330K
Cxm1	ON^b^	His65	Nε	46	29	9
Trp7	Nε	His65	Nδ	21	24	28
Gly4	O	Ser61	Oγ	100	97	80
Trp7	N	Ser61	Oγ	9	5	3
Trp7	O	Leu56	N	71	66	42
Asn11	Oδ	Ala54	N	51	48	37

a Percentage (%) based on a distance between non-hydrogen atoms of less than 3.5 Å.

b Oxygen atom in carboxyl group at the terminal.

**FIGURE 2:**
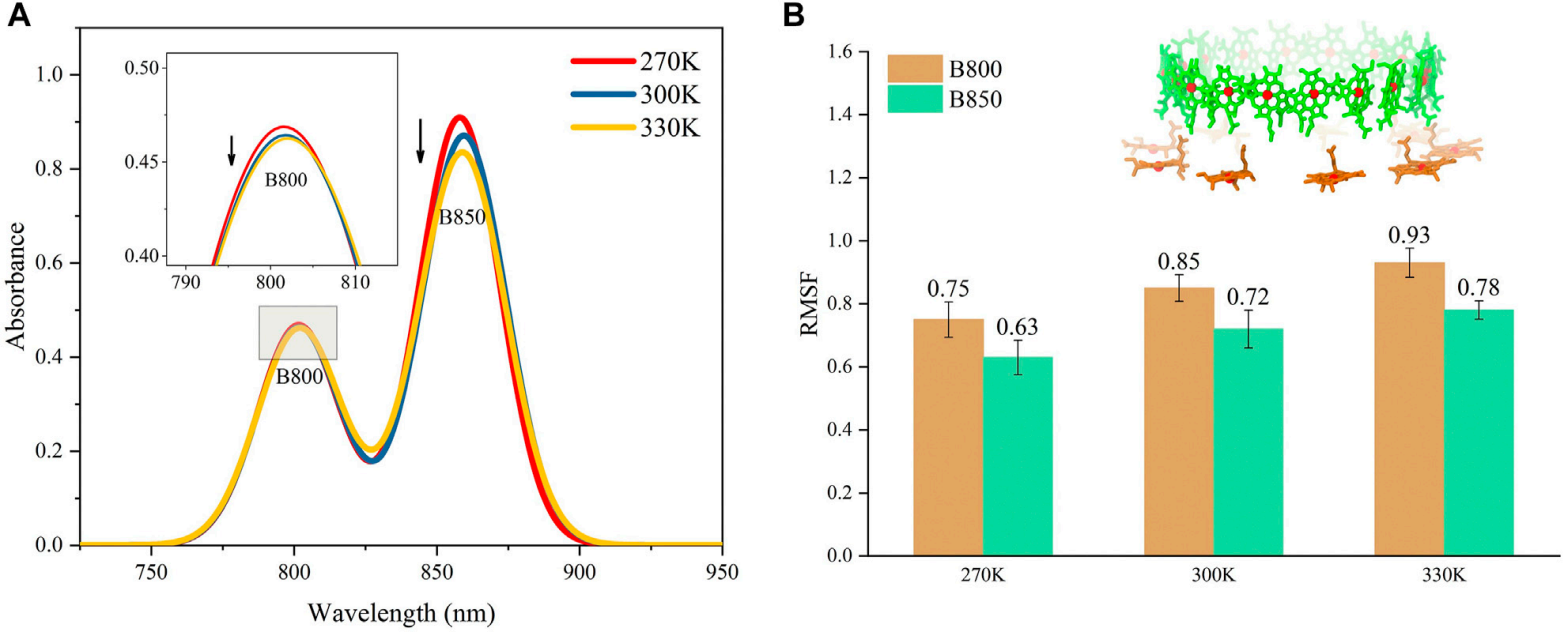
Absorption spectrums for LH2 **(A)** and average conformational variations for B800 and B850 in LH2 **(B)** under different temperatures. The structures for B800 and B850 in LH2 were shown as the insert in **(B)**.

The absorption spectrums of the LH2 were calculated at three different temperatures to further validate the ability of the molecular model to reproduce the thermodynamic properties in MD simulations. As seen in Figure 2, the peaks for the absorption bands of B800 and B850 occurred at 800 and 859 nm under 300 K, respectively, which agreed to those experimentally measured for Rhodoblastus acidophilus. Slight variations for the peaks were observed when temperature varied. More importantly, the absorption amplitudes of B800 and B850 were both decreased as the temperature increasing from 270 to 330 K (Figure 2A). This coordination between the absorption amplitude and temperature agreed to the experimental observations. Previous studies showed that the decrease of the absorption amplitude for LH2 complex was due to the increase of pigment exciton heterogeneity (Alden et al., 1997). As the temperature rose, the increase of the structural motility of pigment molecules was observed (Figure 2B), which was likely to cause the increase of the pigment exciton heterogeneity. Furthermore, the motility for B800 was generally higher than B850 under different temperatures, which would possibly lead to smaller changes in the amplitude of B800 absorption bands.

Collectively, the molecular model of LH2 complex with solvated lipid bilayer in the MD simulations reproduced both dynamic and thermodynamic characters of the complex observed in the experimental measurements. Therefore, the MD simulations were further employed to elucidate the temperature dependency of energy transfers in LH2.

### New Helices Weaken N-Terminus Interactions

**FIGURE 3:**
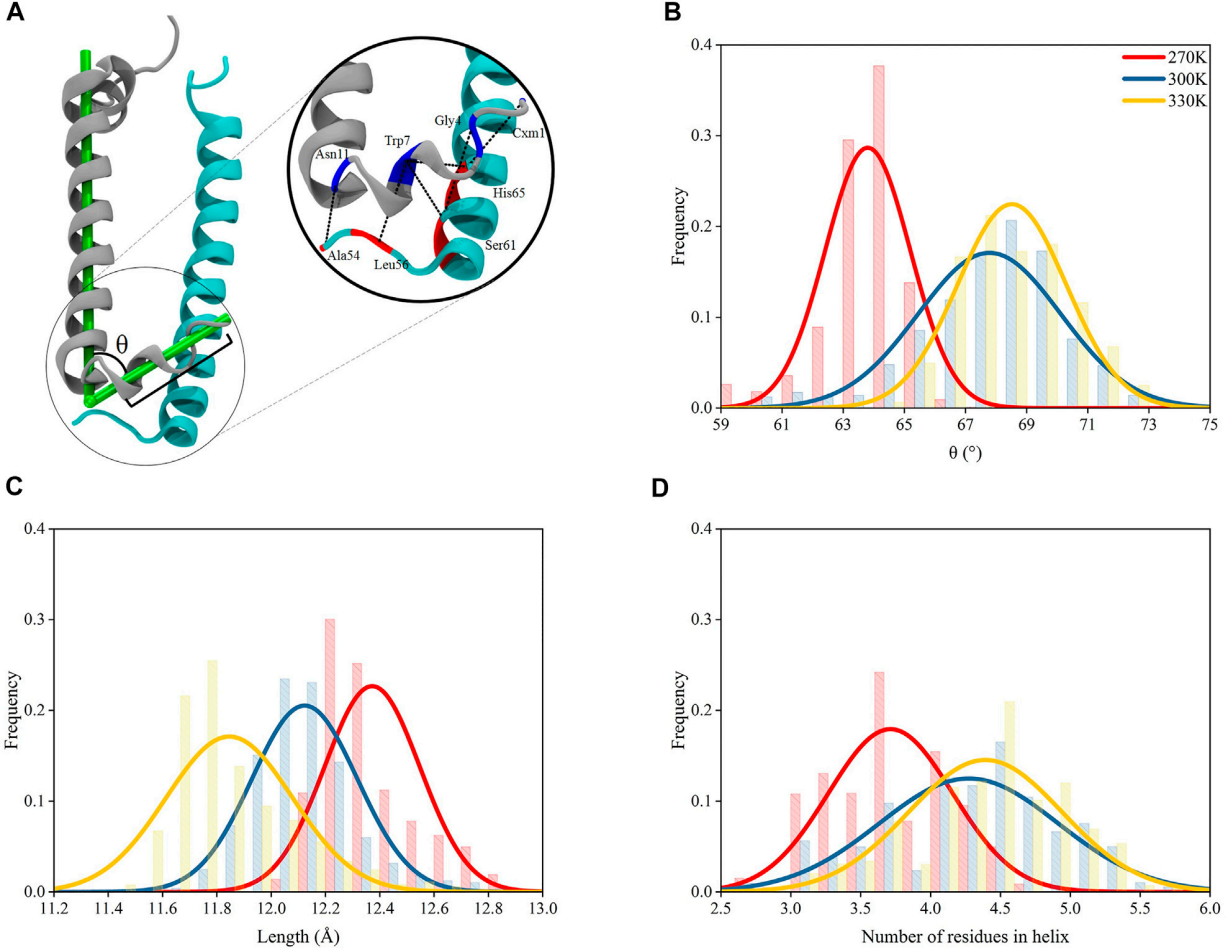
Structural variations for N-terminus of *α* polypeptide helices. **(A)** Interactions at the N-terminus of the heterodimer. The L-angle in the N-terminus of *α* polypeptide helix is defined as the angle between the vector formed by the mass centers of 7th–9th residues and 1st–3rd residues and the vector formed by the mass center of 12th–14th residues and 34th–36th residues, counting from the N-terminus of *α* chain; the length of helices is defined as the distance between the mass center of 7th–9th residues and that of 1st–3rd residues. **(B)** L-angle distributions under 270, 300, and 330 K. **(C)** Distributions of lengths of helices (between the mass centers of 7th–9th residues and 1st–3rd residues) at N-terminus of *α* chains under 270, 300, and 330 K. **(D)** Number of residues in the helical conformation for residue 1–9.

The N-terminus of *α* and *β* polypeptide helices possess high structural flexibility indicated by their B-factor values observed in crystallographic data (Papiz et al., 2003). A recent study has shown that the structures near the terminus of these helices for other complexes in light harvesting system could be affected by temperature changes (Li et al., 2020). Thus, in the present study, the residues near the N-terminus of α and β polypeptide helices were closely monitored during the course of the MD simulations (Figure 3A). Trajectories after 200 ns under each temperature were subjected to structural analyses, whose results were reflected by the average values of those from 9 subunits in the evenly extracted 4,000 frames.

The 9 residues at the N-terminus of *α* polypeptide helices displayed a different orientation comparing to the other residues, which formed an angle in the polypeptide, noted as “L-angle” in this study (Figure 3A). The size of L-angle showed correlations to the temperature. The center for the distribution of its sizes located at 63.8 ± 1.4° under 270 K. When the temperature increased to 300 and 330 K, the center moved to 67.8 ± 2.3° and 68.5 ± 1.5°, respectively (Figure 3B). This observation suggested that the size of the L-angle expanded when the temperature increased. There was also coordination displayed between the average length of the 9 residues at N-terminus and the temperature changes. The average length was 12.4 ± 0.2 Å from MD simulation under 270 K. When temperature increased to 300 and 330 K, the average length reduced to 12.1 ± 0.2 Å and 11.8 ± 0.2 Å, respectively, (Figure 3C). Clearly, the length of these 9 residues decreased with the increase of the temperature. Consequently, the shortening of the length at the N-terminus of *α* polypeptide helices compressed the N-terminus and made more residues form helical structures. As shown in Figure 3D, the average number of residues in the helical conformation at the N-terminus was less than 4 at 270 K, meanwhile this number exceeded 4 at 300 and 330 K. On the other hand, the N-terminus of *β* polypeptide helices have a short free loop and no stable helical structures were formed (Supplementary Figure S4). The inter-helices interactions between *α* and *β* polypeptides were concentrated among the residues near their N-terminus by forming hydrogen bond interactions. Thus, the shortening of the N-terminus and the widening of L-angle in *α* polypeptide helices, together with the flexibility of the short loop at N-terminus of *β* polypeptide helices, reduced the interactions between these two helices. As seen in Supplementary Table S1, the occupancies of those essential hydrogen bonds observed in the crystal structures decreased as the temperature increased with the exception of Trp7-His65, for which the reason of increased occupancy is unclear. These hydrogen bond interactions were essential to the formation of the dimeric *α* and *β* polypeptide helices structures, therefore, the reduction of the occupancies of these hydrogen bonds as the temperature increased showed a temperature-dependent behavior and suggested a consecutive impact on the 9-mer complex of LH2.

### Dissociations of *α* and *β* Polypeptide Helices

**FIGURE 4:**
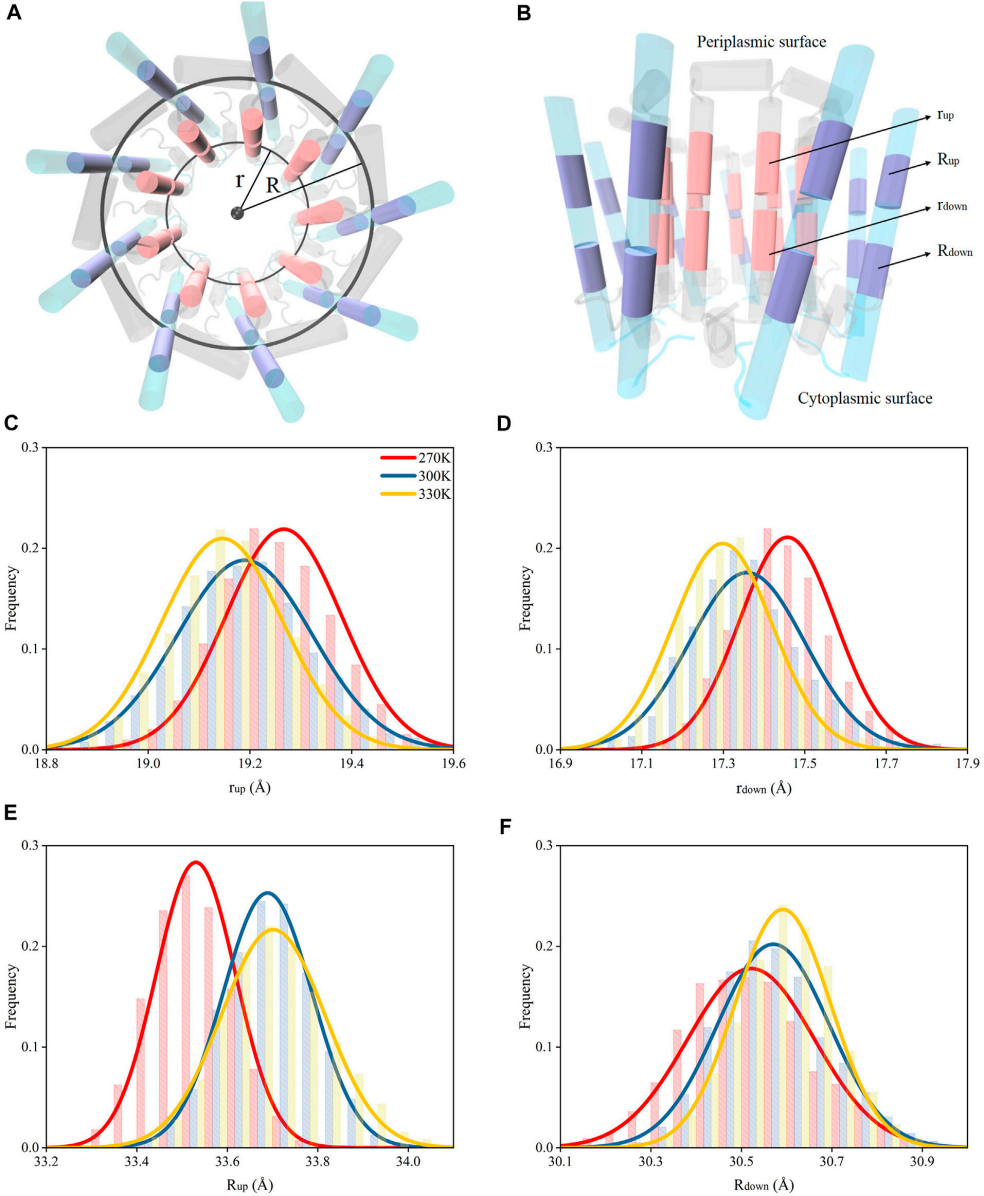
Radii for the rings formed by the polypeptide helices under different temperatures. **(A)** Top view of circular arrangements of *α* (inner) and β (outer) helices in LH2. Radii for *α* and β polypeptide helices were noted as “r” and “R”, respectively. **(B)** Side view of *α* and β polypeptide helices. The circles fit by the mass centers of 26th–32nd (closer to periplasmic surface, colored in pink) and 17th–23rd residues (closer to cytoplasmic surface, colored in pink) in 9 *α* helices are noted as “r_up_” and “r_down_”, respectively. The circles fit by the mass centers of 77th–82nd (closer to periplasmic surface, colored in purple) and 66th–71st residues (closer to cytoplasmic surface, colored in purple) in 9 β helices are noted as “R_up_” and “R_down_”, respectively. Distributions of r_up_ **(C)**, r_down_ **(D)**, R_up_ **(E)**, R_down_ **(F)** under different temperatures.

The dampened interactions between the N-terminus in *α* and *β* polypeptide helices due to the increase of the temperature in each subunit of LH2 could induce a dissociation of the two polypeptides. To investigate, circles were fitted to the rings formed by *α* and *β* polypeptide helices, separately, and their radii were measured. These radii were used to represent the expansion or shrink of the rings. The *α* and *β* polypeptide helices in each subunit were not parallel to each other, or the podetium formed by either *α* or *β* polypeptide helices was not cylindrical. Thus, to more accurately represent the structural changes associated with *α* and *β* polypeptide helices in the LH2 complex, the radii were measured at two locations at trans-membrane helical structures, one near the cytoplasmic surface and the other one near the periplasmic surface. As shown in Figures 4A,B, the rings near the cytoplasmic surface were fitted by the mass centers of residues 26 to 32 and 77 to 82 in *α* and *β* polypeptides, whose radii were measured as r_up_ and R_up_, respectively; those near the periplasmic surface were fitted by the mass centers of residues 17 and 23 and 66 to 71 in *α* and *β* polypeptides with the radii noted as r_down_ and R_down_, respectively.

Both r_up_ and r_down_ reduced as the temperature increased (Figures 3C,D), indicating that the dampened interactions among N-terminus residues could result in shrinking the inner ring formed by *α* polypeptides. Yet, the R_up_ and R_down_ increased when temperature increased. This suggested that weaker interactions among N-terminus residues could cause the expansion of the outer ring formed by *β* chains. Therefore, the nine *α* polypeptides had an opposite moving direction with respect to the nine *β* polypeptides when the temperature increased. In summary, increased temperatures could result in the dissociations of *α* and *β* polypeptides via weakening the intermolecular hydrogen bond interactions.

### Movement of Pigment Molecules

The dissociation of *α* and *β* polypeptide helices could induce positional variations to the pigment molecules because of the stable interactions among pigment molecules and polypeptides in LH2 reported in previous studies (Papiz et al., 2003; Cherezov et al., 2006; Cogdell et al., 2006). In LH2 of purple bacteria, the energies absorbed by RG1 molecules were eventually transferred to B850 molecules, with a portion of those passing through B800 molecules. Therefore, the distances of RG1 and B850 molecules under different temperatures were compared here. Similar to the determination of the dissociation of *α* and *β* polypeptide helices, the relative movements of RG1 and B850 molecules were also measured by the radii of the rings formed by these molecules.

**FIGURE 5:**
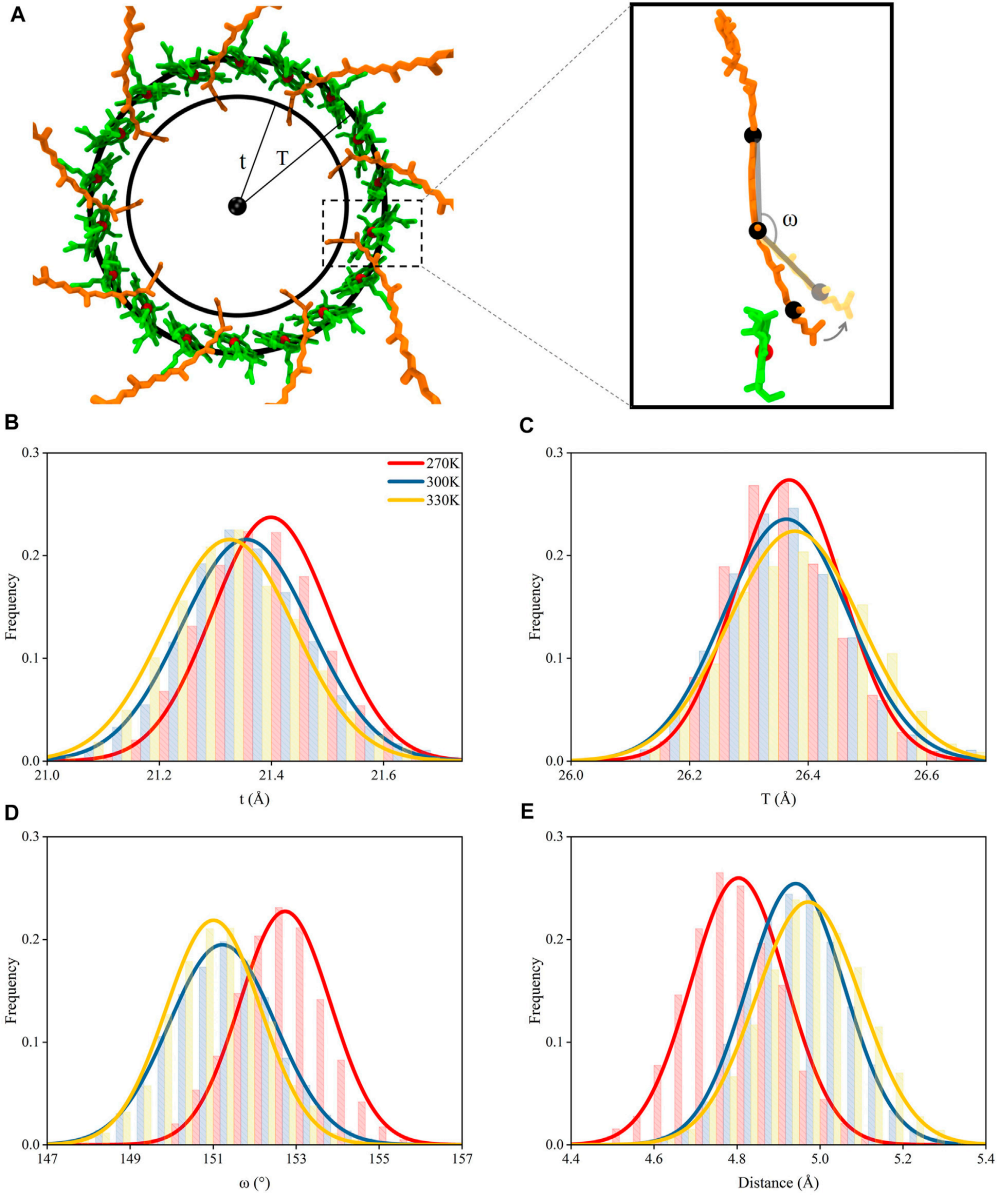
Movements of pigment molecules. **(A)** Top view of rings formed by B850 and the tail of RG1 molecules. The radii of the ring formed by the mass centers of C21–C30 atoms in the tail of RG1 is noted as “*t*” and the radii of the ring formed by the mass center of porphyrin rings in B850 molecules is noted as “T”. The inset displayed the ω angle (measured with mass centers of C9 to C13, C18 to C22, and C26 to C30 atoms, consecutively) change with relation to the distance change between RG1 and B850 molecules **(B)** Distributions of t under different temperatures. **(C)** Distributions of T under different temperatures. **(D)** Distributions of ω angle under different temperatures. **(E)** Distributions of closest distances between RG1 and B850 molecules under different temperatures.

Due to the strong interactions between the tail segment of the RG1 molecules (carbon atoms of C21 to C30, Supplementary Figure S5) and the amino acids in *α* polypeptide helices (Supplementary Table S1), the centripetal movements of *α* polypeptide helices caused by the increase of the temperature reduced the radius for the ring formed by the tail segments of RG1 molecules (noted as “*t*”, Figures 5A,B) and generated a similar centripetal movement for the tail segments of RG1 molecules. Furthermore, these movements bent the RG1 molecules, since the close-to-head segments did not have the same movements. It should also be noted that the RG1 molecules displayed higher flexibility than BChls during the course of the MD simulations (Supplementary Figure S6). Thus, the movements of RG1 molecules could be reflected by the change of this bending angle in RG1 molecules (Figure 5A). As shown in Figure 5D, the bending angle reduced from 153 ± 1.1 at 270 K to 151 ± 1.1 at 300 and 330 K.

The strong π-π stackings among porphyrin rings could maintain the positional stability for B850 molecules, although the B850 molecules showed interactions with residues in both *α* and *β* chains. Thus, the radius for the ring formed by the B850 molecules (noted as “T”, Figure 5C) remained nearly unchanged under different temperatures. Consequently, as the size of the bending angles in RG1 molecules decreased, the tail of the RG1 molecules moved away from the B850 molecules when the temperature increased (Figure 5E). The departing of these two molecules markedly increased the closest distances between these two molecules, which were shown to affect the energy transfers between these two pigment molecules (Koepke et al., 1996; Papiz et al., 2003). As seen in Figure 5E, the closest distance between the tail of RG1 and B850 molecules was 4.8 ± 0.1 Å when the temperature was at 270 K, after the temperature increased, this distance increased to 5.0 ± 0.1 Å. Therefore, the dissociation of *α* and *β* polypeptide helices and the centripetal movements of *α* polypeptide helices caused by the increase of temperature led to the increased distance between RG1 and B850 molecules.

### Coupling Strength Between RG1 and B850 Molecules

The energy transfer capacity between RG1 and B850 could be affected by their electronic coupling strengths, which is essential to the excitonic energy transfers in photosynthetic complexes (Kasha, 2012). Previous studies showed that the electronic coupling strengths were affected by the distances between pigment molecules (Kenny and Kassal, 2016). Thus, the increased distances between RG1 and B850 molecules as a result of the increase of temperature could reduce their coupling strengths. To confirm such speculation, three different methods were employed for calculating the coupling strength of s1-s1 states for RG1 and B850 molecules with their structures extracted from MD simulations under different temperatures.

**FIGURE 6:**
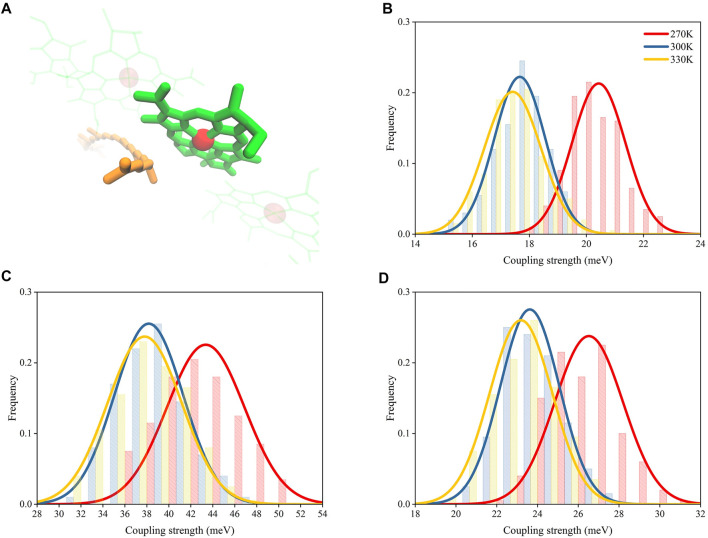
Distributions of coupling strength between RG1 and B850 molecules under different temperatures. **(A)** Representative structures of RG1 and B850. Coupling strengths calculated with PDA **(B)**, BDF **(C)**, and EET analysis **(D)**.

As shown in Figures 6B,D, the coupling strengths computed from structures extracted from MD simulations under 270 K with PDA formulation and the EET analysis in Gaussian16 were 20.5 ± 0.9 and 26.5 ± 1.7 meV, respectively, which are larger than those under 300 K (17.7 ± 0.9 and 23.6 ± 1.4 meV, respectively) and 330 K (17.4 ± 0.9 and 23.1 ± 1.5 meV, respectively). The differences for coupling strengths computed with the BDF program package (Lin et al., 2002) (Figure 6C) appeared to be larger than those computed with the other two methods (43.4 ± 3.5 meV under 270 K, 38.2 ± 3.1 meV under 300 K, and 37.7 ± 3.4 meV under 330 K). It should be noted that a previous study reported smaller values for the calculated RG1-B850 coupling strengths in other species (Tretiak et al., 2000). The inconsistency in the values for the coupling strengths may be caused by their different calculation methods and the innate structural distinctions in RG1-B850 complex.

Although the coupling strength differences computed with the BDF program package (Lin et al., 2002) was nearly twice as those computed with EET analysis (Iozzi et al., 2004) and the PDA formulation, all three computing methods with different level of accuracies appeared to have the same trend for changes of coupling strengths. Such trend obtained independently from three different methods provided confidence towards the proposed temperature-distance-strength relationship.

Because the structures extracted from MD simulations under higher temperatures displayed larger distances between RG1 and B850 molecules (Figure 5E) and these increased distances have shown to reduce the coupling strengths, it can be concluded that the coupling strengths in LH2 complex can be regulated by temperatures via varying distances between pigment molecules.

Moreover, it can also be seen the extent of this regulation was not even in different ranges of temperatures. The temperature changed from 270 to 300 K caused more distance variations for RG1 and B850 (0.14 Å) than that changed from 300 to 330 K (0.06 Å), which coincided with the variations in coupling strength (2.9 and 0.5 meV, respectively, by EET analysis).

In summary, coupling strength between RG1 and B850 molecules decreased, when the temperature increased, was caused by the departing of RG1 and B850 molecules, which was a result of the dissociation of α and β polypeptide helices due to the structural variations at the N-terminus of these helices.

## Conclusion

In the present study, we employed MD simulations and QM calculations for LH2 in purple bacteria under 270, 300, and 330 K, to study the mechanism of temperature variation affecting its energy transfer capability. The molecular model of LH2 in MD simulations reproduced the structural stabilities and essential intermolecular hydrogen bond interactions observed in the experimental data. Variations of the absorption spectrums for LH2 under different temperatures obtained from QM calculations also reproduced those experimentally measured ones, whose changes were attributed to the increased heterogeneity of the exactions for the pigment molecules. These reproductions validated the molecular model of LH2 for further mechanism studies. The MD simulations showed that LH2 adopted the temperature variations through changes in the interactions in protein residues and pigment molecules. In particular, the interactions between the N-terminus of *α* and *β* polypeptide helices were dampened and *α* and *β* polypeptide helices dissociated when temperature increased, resulting the deformation of protein in LH2 complex observed in CD spectrum (Ratsep et al., 2018). The tail of RG1 molecules also moved away from B850 molecules. The coupling strengths computed with different QM methods at different level of theories all suggested the larger distances, as a result of increased temperature, reduced the energy transfer capacity between RG1 and B850 molecules. Based on these observations and results, we proposed a pipeline mechanism for temperature regulating energy transfer in LH2 of purple bacteria during photosynthesis. Higher temperature could promote variations to the structures at the N-terminus of the heterodimer in a subunit, which weakened interactions at N-terminus between *α* and *β* polypeptide helices. As a result, the centripetal and centrifugal movements were discovered for *α* and *β* polypeptide helices, respectively, when temperature increased. The opposite direction movements of polypeptides induced the departing of tail of RG1 molecules from B850 molecules, which, finally, resulted a decrease of their coupling strengths.

The cascading mechanism revealed in our study suggested that the structural changes were resulted from the damped interactions between the N-terminal residues of the α and β polypeptides. Given that the 12 residues at N-terminus of both α and β polypeptide helices are highly conserved in different species (Supplementary Figure S7), especially those forming inter-chain hydrogen bonds, it can be speculated that the proposed regulation mechanism could exist in the protein family of LH2.

It is also worth noting that different ranges of the temperature produced different structural variations for LH2. Three temperatures, 270, 300, and 330 K, were selected to represent the low, room, and high temperature in live physiological conditions, respectively. In general, temperature changing from 270 to 300 K caused larger structural variations than that from 300 to 330 K, i.e., the number of residues in helical structure at N-terminus of *α* polypeptide helices and the distance changes between RG1 and B850 molecules. This phenomenon indicated there might be a threshold for temperature in this regulation. Above this threshold, the changes of structures and energy transfer capability could be less correlated. Furthermore, when temperature increased to a certain value, the dissociation of *α* and *β* polypeptide helices could abolish their interactions and result in the degradation of LH2. This further speculation has been supported by the published CD spectrum, where the structure of LH2 appeared to be significantly altered when temperature increased beyond 340 K (Ratsep et al., 2018).

In summary, MD simulations at micro-second simulation scale unveiled the underlying mechanism for temperature regulating the energy transfer in photosynthesis in LH2 of purple bacteria process via adjusting the position of pigment molecules. The present study paved road for further understanding of energy transfers by LH2 in purple bacteria and shed light on designing temperature adaptive artificial photosynthesis systems.

## Data Availability

The original contributions presented in the study are included in the article/Supplementary Material, further inquiries can be directed to the corresponding authors.
