# Supplementary material for: Bridging Carotenoid-to-Bacteriochlorophyll Energy Transfer of Purple Bacteria LH2 With Temperature Variations: Insights From Conformational Changes
Source: Front Chem. 2021 Oct 4;9:764107. doi: 10.3389/fchem.2021.764107 (PMC8521103; doi:10.3389/fchem.2021.764107)
Supplement: Supplementary file 1 [file DataSheet1.pdf]

Supplementary Material

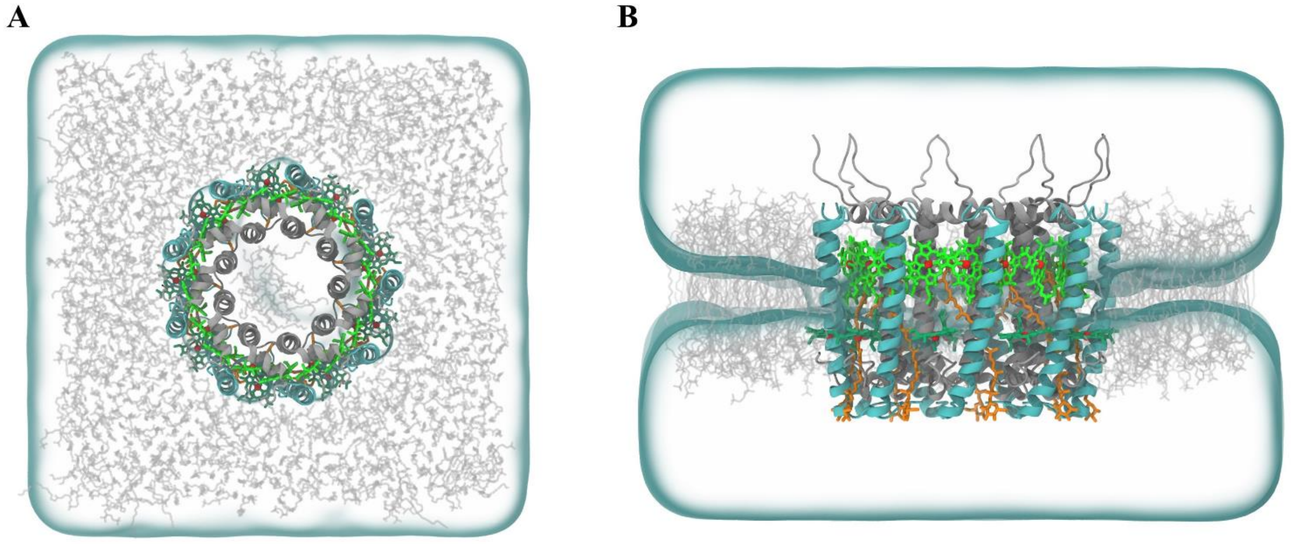

Figure S1. Purple bacteria LH2 complex embedded in the POPC membrane (**(A)**: top view; **(B)**: side view). α and β polypeptide helices are color in silver and cyan, respectively. Pigment molecules are colored in dark green (B800), bright green (B850), and orange (RG1). The phytol portion in BChl was not displayed.

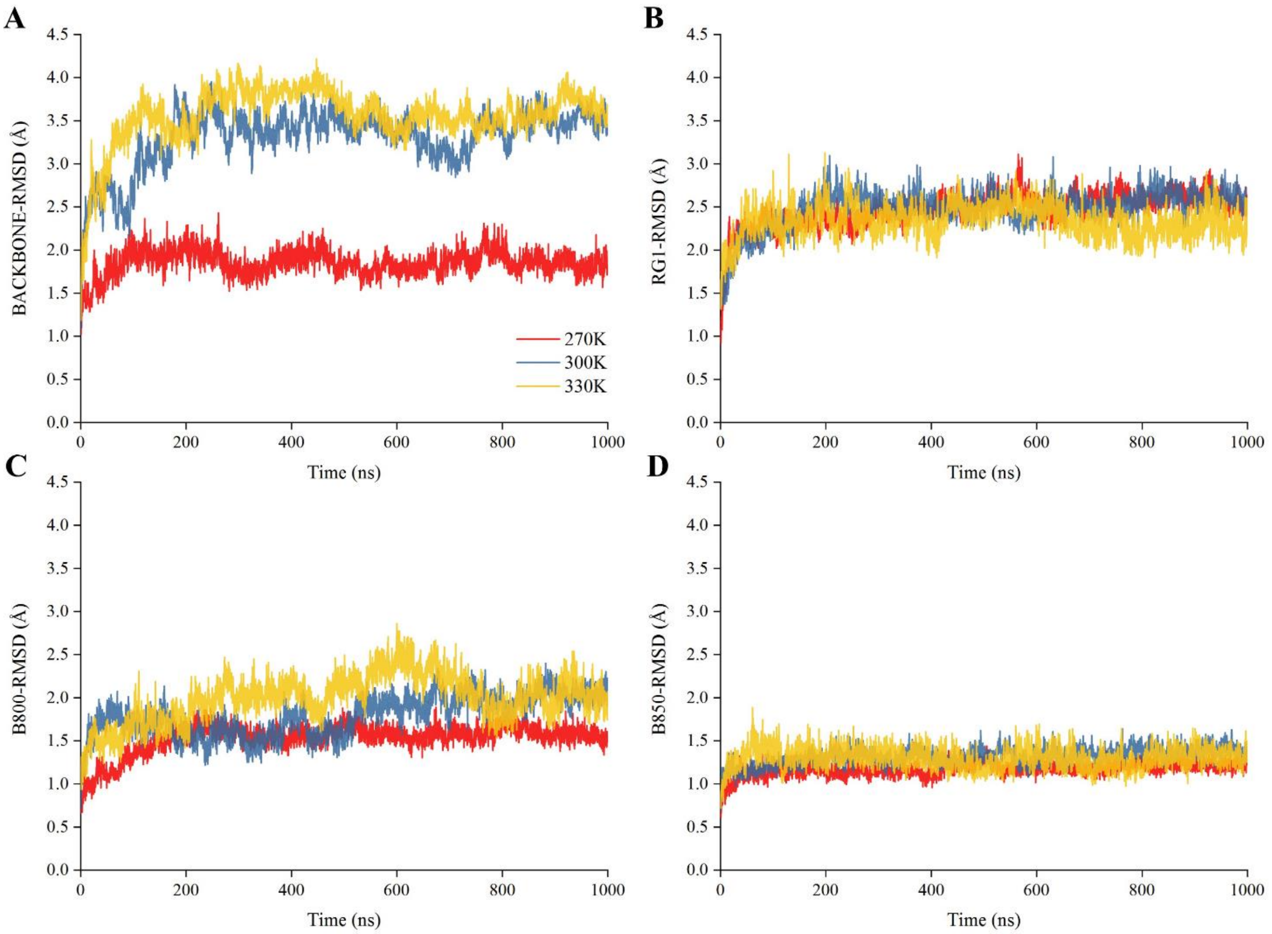

Figure S2. Time dependent RMSDs from the starting structure during a 1μs molecular dynamics trajectory for LH2 at different temperatures (**(A)**: protein backbones; **(B)**: RG1; **(C)**: B800; **(D)**: B850). The RMSD of protein backbone is calculated by the Cα,C,N and O atoms of protein, RG1 is obtained by non-hydrogen atoms, and BChl are derived from the heavy atoms of the porphyrin ring.

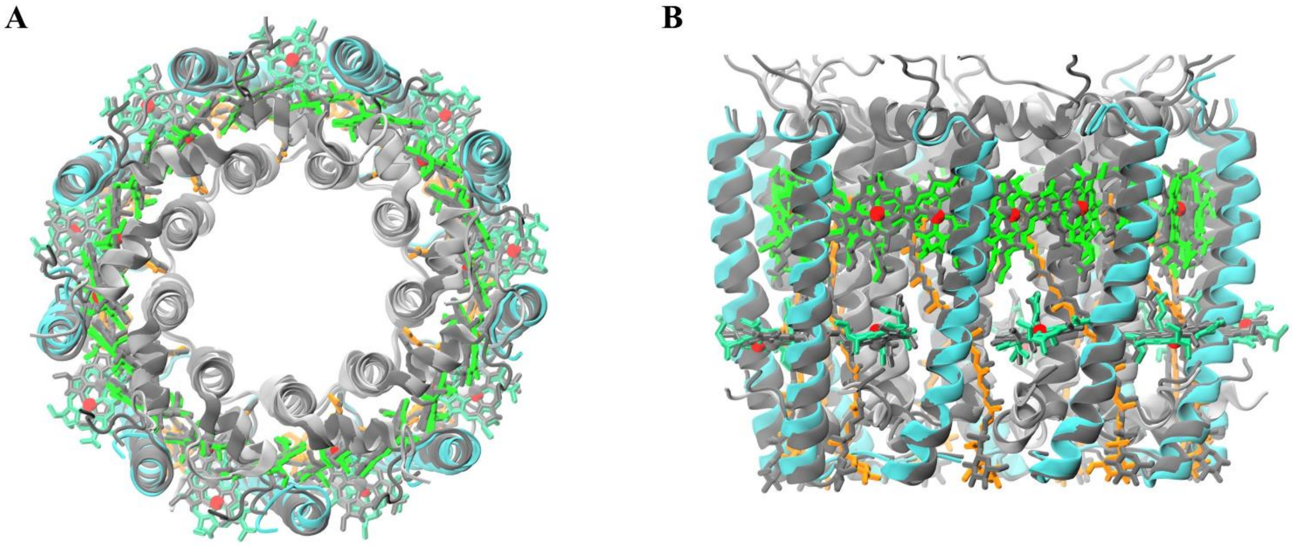

Figure S3. The superposition of the starting structure and the representative structure of the MD-trajectory at physiological temperature. The view of Figure **(A)** is perpendicular to the plane of the fully hydrated POPC lipid bilayer in which the system was embeded, and the view of Figure **(B)** is parallel to plane. The starting structure is represented in gray, and the representative structure color refers to Figure 1.

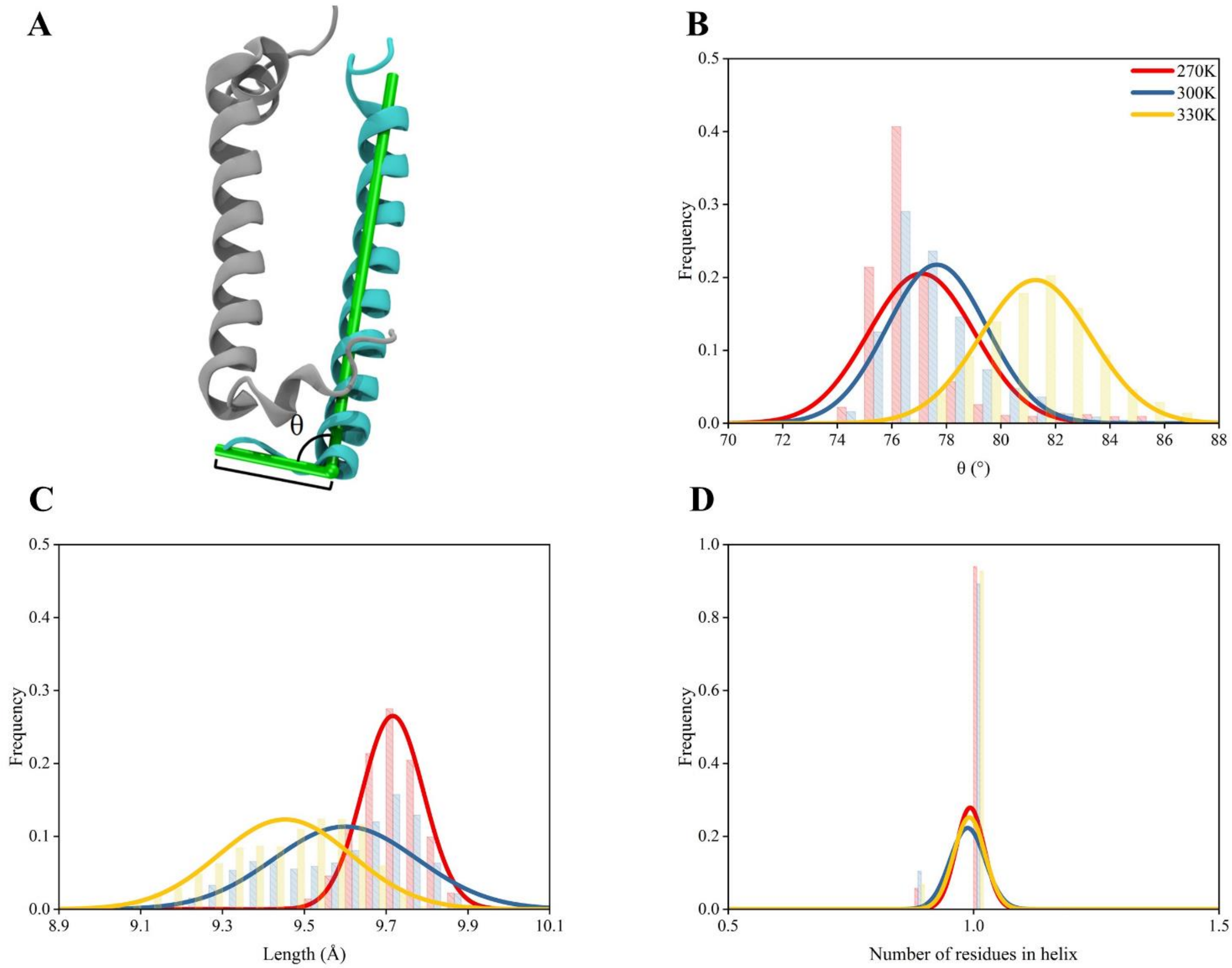

Figure S4. Structural variations for N-terminus of β polypeptide helices. (A). Interactions at the N-terminus of the heterodimer. The L-angle in the N-terminus of β polypeptide helix is defined as the angle between the vector formed by the mass centers of 56th-57th residues and 53th-54th residues and the vector formed by the mass center of 59th-61th residues and 84th-86th residues, counting from the N-terminus of α chain; the length of helices is defined as the distance between the mass center of 53th-54th residues and that of 56th-57th residues. **(B)**. L-angle distributions under 270, 300, 330K. **(C)**. Distributions of lengths of helices (between the mass centers of 53th-54th residues and 56th-57th residues) at N-terminus of β chains under 270, 300, 330K. **(D)**. Number of residues in the helical conformation for the first 5 residues.

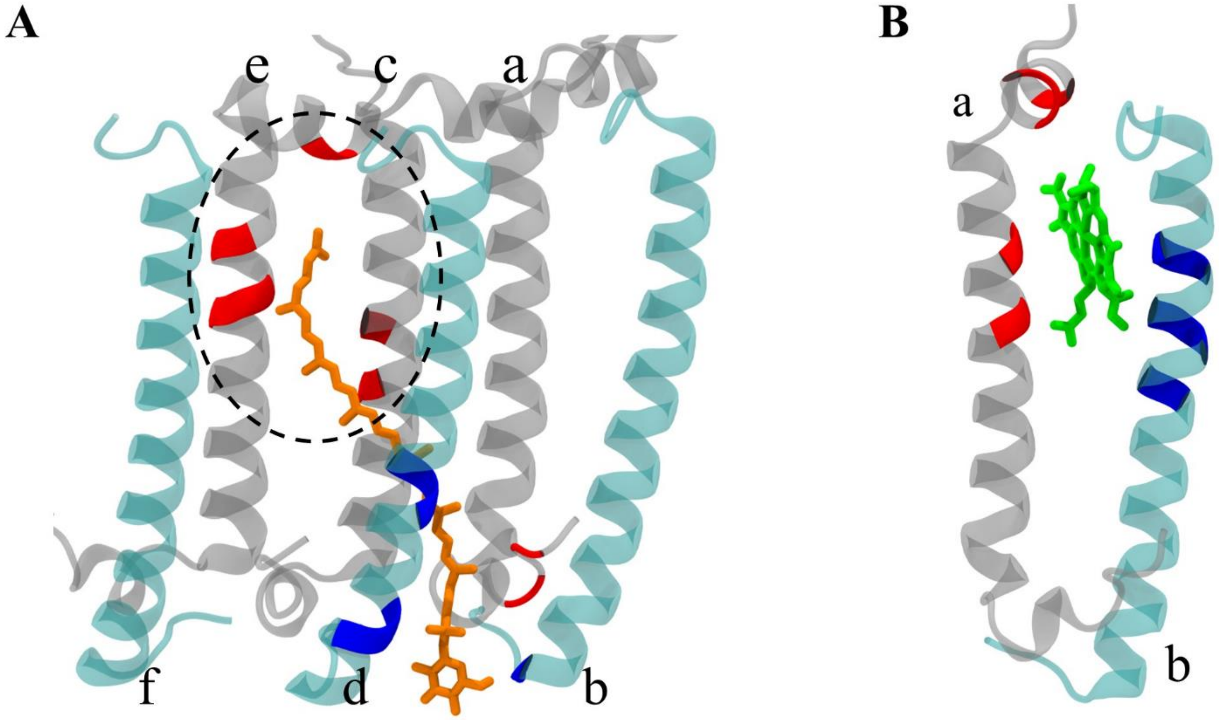

Figure S5. The interaction of RG1 and α-B850 with protein. Figure **(A)** shows the interaction between RG1 and the protein, a, c, and e represent the three α chains of the protein, b, d, and f represent the three β chains of the protein, and the black dotted line indicates the interaction between the tail of RG1 and the protein; Figure **(B)** shows the interaction between α-B850 and protein, a and b represent the alpha chain and β chain of the protein, respectively. The color of protein and pigment molecules refers to Figure 1, in which α-chain residues that interact with pigment molecules are shown in red, such a β-chain residues are shown in blue. The interaction between residues is calculated using LigPlot+ (Laskowski and Swindells, 2011), please refer to Table-S1 for the residue index.

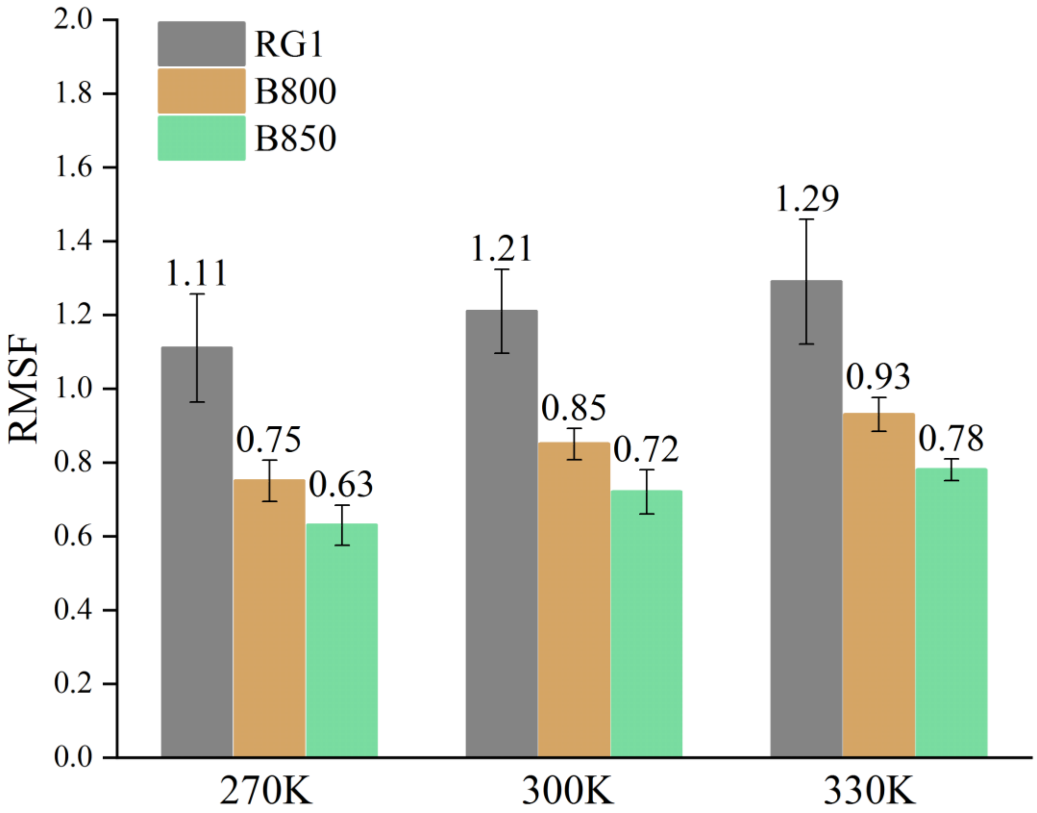

Figure S6. Average conformational variations for different pigment molecules in LH2 under different temperatures.

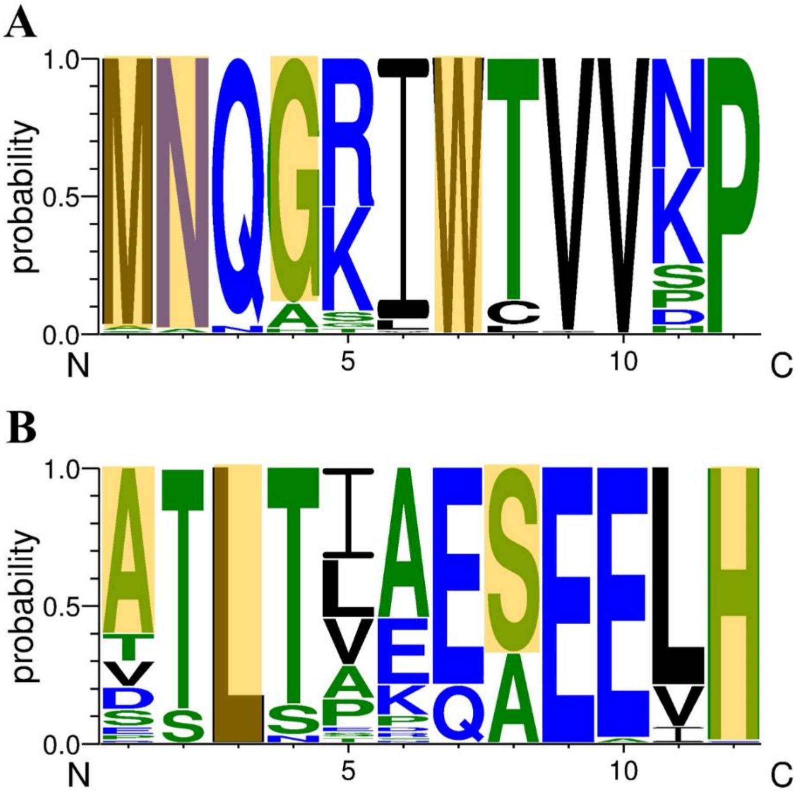

Figure S7. Conservation analysis of N-terminal residues in α and β chains. Figure **(A)** represents the conservation of N-terminal residues in the α chain; Figure **(B)** represents the conservation of the N-terminal residues in the β chain. The Hydrophilic residues are represented by blue, the Neutral residues are represented by green, and the Hydrophobic residues are represented by black. The yellow box indicates the residue that forms a hydrogen bond at the N-terminal.(We first used the Basic Local Alignment Search Tool(BLAST) program (Altschul et al., 1990) to search for the similarity sequence of the N-terminal residues, then used ClustalX2 (Larkin et al., 2007) to perform multiple sequence alignment on the obtained sequences, and finally generate the sequence logos by WebLogo3 (Crooks et al., 2004).)

Table S1. The protein residues that interact with RG1 or B850.

RG1		B850	
α helices	β helices	α helices	β helices
a-Gln3	b-Ala5	a-Ala27	b-Phe22
a-Lys5	d-Glu10	a-His31	b-Ala29
c-Val23	d-Leu11	a-Trp40	b-His30
c-Ile26	d-Gly18	a-Phe41	b-Leu25
e-Ala27	d-Thr19	a-Trp45	b-Ala26
e-Ile28			
e-His31			
e-Trp45			

Both the alpha chain and the beta chain are numbered from the N-terminus and the first residue number is set to 1;

a-e correspond to the protein chain numbers in Figure S5.

References

Altschul, S.F., Gish, W., Miller, W., Myers, E.W., and Lipman, D.J. (1990). Basic Local Alignment Search Tool. *J. Mol. Biol.* 215**,** 403-410. doi:10.1016/S0022-2836(05)80360-2

Crooks, G.E., Hon, G., Chandonia, J.M., and Brenner, S.E. (2004). WebLogo: A Sequence Logo Generator. *Genome Res.* 14**,** 1188-1190. doi:10.1101/gr.849004

Larkin, M.A., Blackshields, G., Brown, N.P., Chenna, R., Mcgettigan, P.A., Mcwilliam, H., Valentin, F., Wallace, I.M., Wilm, A., Lopez, R., Thompson, J.D., Gibson, T.J., and Higgins, D.G. (2007). Clustal W and Clustal X Version 2.0. *Bioinformatics* 23**,** 2947-2948. doi:10.1093/bioinformatics/btm404

Laskowski, R.A., and Swindells, M.B. (2011). LigPlot+: Multiple Ligand-Protein Interaction Diagrams for Drug Discovery. *J. Chem. Inf. Model.* 51**,** 2778-2786. doi:10.1021/ci200227u
